# Supplementary material for: The Role of LncRNAs in Radio- and Chemoresistance of Glioblastoma: Prognostic or Therapeutic?
Source: Curr Oncol. 2025 Sep 27;32(10):539. doi: 10.3390/curroncol32100539 (PMC12562516; doi:10.3390/curroncol32100539)
Supplement: Supplementary file 1 [file curroncol-32-00539-s001.zip › curroncol-3835106-supplementary.pdf]

Table S1. lncRNA , miRNA sponge, pathway involved.

**lncRNA , miRNA sponge, pathway involved**						
lncRNA	miRNA	pathway	Tumor Suppressor	Oncogene	Drugs	PMDI
**LINC01410**	miR-370-3p	PTEN/AKT		X		34435542
**LINC01123**	miR-151a	CENPB		X		34519373
**LINC00021**		P21/EZH2		X		32449315
**LINC00511**	miR-126-5p	Wnt/β-catenin		X		34328678
**LINC00883**	miR-136	NEK1		X		35083134
**LINC00461**	miR-216	AQP4		X	R	33817241
**LINC00520**		STAT3/ LIN28B		X		35945579
**LINC01057**		NF-κB		X		33130316
**LINC00174**	miR-138-5p	SOX9 axis		X		31713817
**Linc00942**		SOX9 STAT3/P300 axis		X		39342418
**LINC00473**		CREB/CEBPα/MGMT		X		37981063
**LINC01956**		*MGMT*		X		39356744
**LINC00957**	miR-17-5p	*NPNT*		X		39430842
**HOTAIR**	miR-519a-3p	RRM1		X		32721218
		CALCOCO1 and ZC3H10		X		31953347
	miR-526b-3p	EVA1		X		35418162
	miR-125	HK2		X		32279420
		HOTAIR/EZH2		X	EPIC-0628	38490327
	miR-214-3p	HOTAIR/miR-214-3p/β-catenin network		X	meta-troxate	39482401
**MIR155HG**		Wnt/β-catenin/PTBP1		X		32529543
**TPTEP1**	miR-106a-5p	P38 MAPK	X		R	33173989
**TP53TG1**	miR-524-5p	RAB5A		X	R	32762546
**BC200**	miR-218-5p			X		32784466
**NCK1-AS1**	miR-22-3p	IGF1R		X		32887025
	miR-137	TRIM24		X		31750728
**KCNQ1OT1**	miR-761			X		32897512
**linc-RA1**		H2Bub1/ USP44				32934196
**H19**	miR-93a	ATG7 pituitary	X			32946927
**H19 and HOXD-AS2**	miR198	SMAD			TGF-β1	
		CREB1		X		34159190
		ZEB1				
**NEAT1**	let-7g-5p	MAP3K1		X		33057597
	(miR)-324-5p	KCTD20		X		33982764
	miR-23a-3p	GLS glutaminase (medulloblas-toma)		X		35313796

**LIFR-AS1**	miR-4262	NF-κB	X			33070767
**MSC-AS1**	miR-373-3p	CPEB4		X		33106913
**CRNDE**	miR-29c-3p			X		31753063
		PI3K/Akt/mTOR<br>ABCG2 expression		X		34454479
**SNHG12**	miR-129-5p	MAPK1 and E2F7		X		32039732
**CCAT2**	miR-424	Chk1				32110042
**PSMB8-AS1**	miR-22-3p			X		32151711
**EPIC1**		Cdc20		X		32322669
**SOX2OT**		Wnt/β-catenin		X		32439916
**HSPA7**		SPP1<br>YAP1 and LOX				
**JPX**		FTO/PDK1				
**HOTAIRM1**		TGM2		X		34584066
**lnc-TALC**		tumor-associated macrophages (TAM) and p38 MAPK				34667108
**TMEM161B-AS1**	miR-27a-3p-	FANCD2/CD44				
**FOXD2-As1**		EZH2				
**lncRNA-MVIH**	miR-302a			X		33438023
**HCP5**	miR-128					
**OIP5-AS1**		IGF2BP2		X		34132932
**RBM5-AS1**		SIRT6		X		34225779
**ATXN8OS**		ADAR/GLS2				
**XLOC013218**		PIK3R2 XLOC/Sp1/PIK3R2/PI3K/ AKT				
**HCG11**	microRNA-144	Cox2				
**MIR222HG**		SP1		X		37351164
**PDIA3P1**		C/EBPβ-MDM2				
**TUG1**		EZH2	X			34036375
**MAFG-AS1**	miR-642a-5p	Notch1		X		36214714
**ZBED3-AS1**		THBD	X			36462734
**HOXA-AS2**	miR-302a-3p	IGF1		X		36479381
**KCNQ1OT1**	miR-761	PIM and c-MYC				32897512
**TUSC7**		PTEN				
**OIP5-AS1**	miR-129-5p	IGF2BP2				34132932
**MUF**	miR-34a	SNAIL1		X	TGF-β-	35223453
**PSMG3-AS1**		c-MYC				
**MIR210HG**		IGFBP2		X		34897892
**DANCR**		FOXO1		X		35141227
**FOXD3-AS1**	miR-128-3p	WEE1 G2 checkpoint kinase axis		X		35191808
**ARFRP1 and RUSC2**		AMPK, AKT, mTOR, and TGF-β signaling		X		37720935
**MAGI2-AS3**		AKT	X			37551766

DARS1-AS1		*DARS1-AS1*/YBX1		X		37540752
**PVT1**		JAK/STAT		X		37202742
**ZBED3-AS1**						
**CCAT2**	miR-424	Chk1		X		32110042
**TMEM161B-AS1**	miR-27a-3p	FANCD2/CD44		X		34689169
**RMRP**		RMRP/ZNRF3 axis and Wnt/β-catenin		X		34657141
**JPX**		FTO/PDK1 axis		X		34390075
**HCP5**	miR-128			X		33994812
**PDIA3P1**		C/EBPβ drug combination NEF		X		35836243
**RNA HCG11**	miR-44COX-2			X		35818213
**XLOC013218**		The XLOC/Sp1/PIK3R2/PI3K/AKT axis		X		35637600
**ATXN8OS**		ADAR/GLS2 pathway	X			35460867
**PSMG3-AS1**		c-Myc		X		35380741
**TP73-AS1**			X			37452786
